# Out-of-hours services and end-of-life hospital admissions: a complex intervention systematic review and narrative synthesis

**DOI:** 10.3399/BJGP.2021.0194

**Published:** 2021-09-07

**Authors:** Evie Papavasiliou, Sarah Hoare, Ben Bowers, Michael P Kelly, Stephen Barclay

**Affiliations:** PELiCam Palliative and End of Life Care Group, Primary Care Unit, Department of Public Health and Primary Care, University of Cambridge, Cambridge.; PELiCam Palliative and End of Life Care Group and THIS Institute (The Healthcare Improvement Studies Institute), Department of Public Health and Primary Care, University of Cambridge, Cambridge.; PELiCam Palliative and End of Life Care Group, Primary Care Unit, Department of Public Health and Primary Care, University of Cambridge, Cambridge.; PELiCam Palliative and End of Life Care Group, Primary Care Unit, Department of Public Health and Primary Care, University of Cambridge, Cambridge.; PELiCam Palliative and End of Life Care Group, Primary Care Unit, Department of Public Health and Primary Care, University of Cambridge, Cambridge.

**Keywords:** general practice, out-of-hours, admission, hospitalisation, palliative care, terminal care

## Abstract

**Background:**

Out-of-hours (OOH) hospital admissions for patients receiving end-of-life care are a common cause of concern for patients, families, clinicians, and policymakers. It is unclear what issues, or combinations of issues, lead OOH clinicians to initiate hospital care for these patients.

**Aim:**

To investigate the circumstances, processes, and mechanisms of UK OOH services-initiated end-of-life care hospital admissions.

**Design and setting:**

Systematic literature review and narrative synthesis.

**Method:**

Eight electronic databases were searched from inception to December 2019 supplemented by hand-searching of the *British Journal of General Practice*. Key search terms included: ‘out-of-hours services’, ‘hospital admissions’, and ‘end-of-life care’. Two reviewers independently screened and selected articles, and undertook quality appraisal using Gough’s Weight of Evidence framework. Data was analysed using narrative synthesis and reported following PRISMA Complex Intervention guidance.

**Results:**

Searches identified 20 727 unique citations, 25 of which met the inclusion criteria. Few studies had a primary focus on the review questions. Admissions were instigated primarily to address clinical needs, caregiver and/or patient distress, and discontinuity or unavailability of care provision, and they were arranged by a range of OOH providers. Reported frequencies of patients receiving end-of-life care being admitted to hospital varied greatly; most evidence related to cancer patients.

**Conclusion:**

Although OOH end-of-life care can often be readily resolved by hospital admissions, it comes with multiple challenges that seem to be widespread and systemic. Further research is therefore necessary to understand the complexities of OOH services-initiated end-of-life care hospital admissions and how the challenges underpinning such admissions might best be addressed.

## INTRODUCTION

The out-of-hours (OOH) period (nights, weekends, and bank holidays) comprises 63% of the week in the UK, when normal in-hours primary care services are not available. End-of-life care in the community is an important and challenging aspect of OOH provision, and hospital admissions for patients at the end of life are controversial.^1^^–^^5^ Recent research has challenged perceptions of hospital admissions for patients close to the end of life as inappropriate, preventable, or avoidable,^6^^–^^8^ and has highlighted that at times hospital is the only place where care is reliably, safely, and urgently available.^9^^,^^10^

The challenges facing GPs, ambulance staff, nurses, and other OOH providers in the delivery of high-quality end-of-life care are significant and multifaceted. These include access to patient information;^11^ meeting the clinical needs of patients often very close to death;^12^ lack of confidence in providing end-of-life care;^13^^,^^14^ a potentially awkward fit between end-of-life care and services’ wider remit of urgent care;^15^^–^^17^ uncertainties of prognostication; and decisions about whether the patient’s condition is potentially reversible with hospital treatment or if they are best kept at home for symptomatic relief and care.^15^ Hospital care is at times the best option for patients at the end of life to reliably obtain urgently needed care OOH.^15^ Only some end-of-life-care related OOH calls, however, lead to hospital admissions.^12^ It is unclear which issues or combination of issues lead OOH clinicians to initiate hospital care and when patients are best kept at home. The aim of this study was to review the literature concerning the mechanisms (the components of the system that initiate admissions), the circumstances (the context of and reasons for admissions), and the processes (the actions and steps through which admissions are instigated) involved.

For the purposes of this review end-of-life care was defined as the care of patients with advanced incurable disease and an anticipated prognosis of ≤12 months of life. The term OOH providers is used to refer to all services providing access to health care for patients at night, weekends, or bank holidays. In this review, all end-of-life care hospital admissions occurring OOH, whatever the outcome of the admission, were included, and these were not limited to admissions in which patients necessarily died in hospital after admission.

The following six questions were addressed:

- Which patients are admitted?
- What are the mechanisms of these admissions?
- Which OOH providers arrange these admissions?
- How frequently are these admissions arranged?
- Why are these admissions arranged?
- What are the processes of these admissions?

**Table:** How this fits in

Out-of-hours (OOH) end-of-life care hospital admissions are a concern for patients, families, clinicians, and policymakers. Little is known about the mechanisms, processes, and circumstances under which such admissions occur. This review found that admissions occur to address clinical needs, unavailability or discontinuity of care, and patient or carer distress. They are initiated by a variety of OOH providers and services, indicating the issues identified are widespread and systemic. Existing evidence, however, is scarce, and further research is required to understand why these admissions occur and how the issues identified might best be addressed.

## METHOD

### Data sources

MEDLINE Complete, Embase Excerpta Medica, Cochrane Database of Systematic Reviews, PsycINFO, CINAHL Complete, Social Care online, Web of Science Core Collection, and Scopus were systematically searched from inception to December 2019. Database searches were undertaken by the information scientist member of the review team on 16 December 2019. A hand-search of the *British Journal of General Practice*, identified as the journal in which most relevant publications occurred, and citation searches of included publications were undertaken by the first author in May 2020.

### Inclusion and exclusion criteria

Inclusion and exclusion criteria were developed using the PICOTS framework (Population, Intervention, Comparator/Context, Outcome, Timing, and Setting) (Box 1).^18^

**Box 1.** Inclusion and exclusion criteria

**PICOTS**	**Included**	**Excluded**
**Population**	Patients: Aged>18 years With advanced incurable disease and/or with an anticipated prognosis of a year or less	Patients: Children/adolescents With early-stage or curable disease Unexpected or sudden death
**Intervention**	Out-of-hours services: NHS 111 999 ambulance care Community nursing Out-of-hours general practice Out-of-hours specialist provision (palliative care)	Out-of-hours services: Out-of-hours medicines Out-of-hours dental emergencies
**Comparator**	Not applicable	—
**Context**	Published output: Articles of any design reporting original empirical findings (either as the study focus or an outcome measure) Focusing on UK health care	Published output: Book chapters, letters, comments, and editorials Focusing on international health care
**Outcome**	Hospital admissions: Any department of a hospital	Hospital admissions: Psychiatric care department
**Timing**	No time restriction	—
**Setting**	Home/community care Nursing/care home Long-term care facilities Prison	Hospice

### Search strategy

Preliminary searches, although highly sensitive, lacked specificity, as no publications were identified that directly addressed all three target domains (OOH services, hospital admissions, and end-of-life care). The search strategy was therefore revised to include search terms for both end-of-life care and palliative care as these were often used interchangeably in the studies identified. Searches were also modified to focus on two target domains: end-of-life care and hospital admissions (Dataset I) *or* end-of-life care and OOH (Dataset II), with OOH and hospital admissions excluded as the literature identified was large and diffuse. The final database search for MEDLINE can be found in Supplementary Box S1. The information scientist member of the team advised that place names were also included to maximise identification of studies.

**Figure 1.**
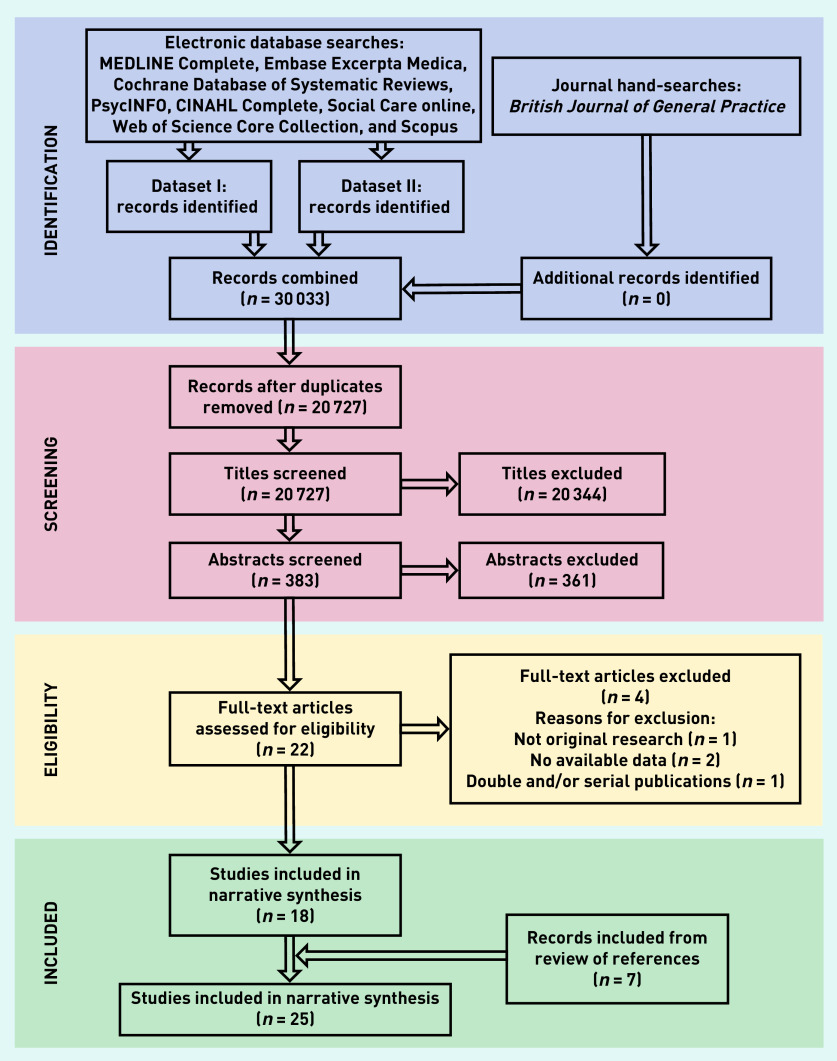
*PRISMA flow chart: selection process for systematic review on end-of-life care hospital admissions arranged by out-of-hours services. Dataset I = end-of-life care and hospital admissions. Dataset II = end-of-life care and out-of-hours.*

Search results were imported to EndNote (version X9) and de-duplicated. The PRISMA flow diagram for study selection is shown in Figure 1.

### Study selection and data extraction

Title screening was undertaken by the first author and abstract screening by the first author and one other author independently, with disagreements resolved by discussion. Full texts of potentially eligible publications were assessed by the first author and reviewed by two or more authors if there was uncertainty. Data were extracted into a review-specific data extraction form provided in Supplementary Table S1.

### Quality appraisal

Gough’s Weight of Evidence (WoE) framework was used to assess the quality and relevance of included publications.^19^ This framework rates the internal validity of the study (WoE A), appropriateness of study design to the review aims (WoE B), and the focus or relevance of the study to the review aims (WoE C). Each of the three domains is individually scored on a scale from 1 (low) to 3 (high), and combined to provide an overall judgement (mean score of A, B, and C) to generate an overall assessment of study quality and relevance (WoE D). WoE appraisal was carried out independently by three authors, with disagreements resolved by discussion. Publications assessed as being of high WoE were considered more credible and relevant and were given priority during data synthesis.^19^^,^^20^ Given the scarcity and diffuse nature of the evidence identified, publications assessed as low WoE were also included.

### Data synthesis

Data synthesis used a narrative approach,^20^^,^^21^ selected for its potential to assess and synthesise heterogeneous and complex evidence in a rigorous and replicable way.^22^^,^^23^ A thematic approach was employed for review questions where sufficient data was available whereas a descriptive approach was adopted for questions with limited available evidence. Reporting followed the PRISMA Complex Interventions extension statement and checklist.^24^^,^^25^

### Patient and public involvement

A patient and public involvement advisory group of six people with experience of hospital admissions towards the end of life met twice, initially advising on refinement of review questions and later commenting on emerging findings. The review protocol was registered with PROSPERO (registration number: 42019156827).

## RESULTS

Database searches retrieved 30 033 records. Following de-duplication and title and abstract screening, 22 articles were assessed in full text for eligibility. Four were excluded and seven additional articles were identified from hand-searching and reference lists of eligible articles, leading to a final total of 25 included publications (Figure 1).^1^^,^^10^^,^^12^^,^^15^^,^^26^^–^^46^

### Characteristics of included studies

The included publications comprised 23 original articles and 2 project reports, using qualitative (*n* = 9), quantitative (*n* = 10), and mixed-methods designs (*n* = 6). Studies were conducted in the UK: UK-wide (*n* = 2), England (*n* = 13), Scotland (*n* = 7), England and Scotland (*n* = 2), and Northern Ireland (*n* = 1). Articles were published between 2002 and 2020. Only one article was dated before the 2004 UK introduction of the new contract through which responsibility for commissioning and providing OOH care passed from GPs to primary care trusts.^26^ Supplementary Table S2 summarises the included publications.

### Focus and nature of available evidence

Publications varied considerably in terms of their focus and the nature of the evidence reported with respect to the three dimensions of this review. Most presented either ‘indirect evidence’ (data extracted was interpreted, for example ‘68% of hospital admissions occurred within working hours’ implies that 32% occurred OOH) and/or ‘generalised evidence’ (data extracted was implied, for example ‘68% of advanced cancer patients were admitted to hospital’ implies some were admitted OOH). See Supplementary Table S3 for detailed mapping of included publications based on focus and nature of available evidence. Results are presented with priority being given to those review questions for which the most evidence is available.

### Reasons for OOH hospital admissions at the end of life

In the 19 publications addressing reasons for OOH hospital admissions at the end-of-life review question,^1^^,^^10^^,^^15^^,^^27^^–^^42^ eight themes were identified, grouped under thematic areas of participants, structures and processes, and clinical factors (see Box 2).

**Box 2.** Circumstances leading to out-of-hours hospital admissions for patients receiving end-of-life care

**Thematic area**	**Themes**	**Subthemes**
**Participants**	Patients	Patient needs or wishes
	Caregivers	Carer inexperience of death Carer preferences Carer burnout Carer uncertainty Carer breakdown
	Both	Patient or carer distress Patient or carer anxiety

**Structures and processes**	Policy	Lack of advance care planning Concerns about timely access to anticipatory medication
	Organisation	Unavailability or limited capacity of alternative services Lack of systems in place Lack of availability or accessibility of patient information Lack of flexibility (fixed structure) Complexities of workforce management Lack of continuity of care Lack of time to assess and address needs
	Provider	Lack of training Poor communication Inability to provide care Unfamiliarity with the patient Feeling of not performing their duties Fear of professional repercussions

**Clinical factors**	Assessment or diagnosis	Clinical or diagnostic uncertainty Complexity of the situation
	Symptom management	Treatment of pain or other physical symptoms Complication of treatment or failure of initial treatment
	Precaution or follow-up	As a precaution Abnormal laboratory results Further investigation

In terms of participants, reported circumstances related predominantly to informal caregivers, associated with their preferences, inexperience of death, and feelings of uncertainty, burnout, or emotional breakdown.^1^^,^^10^^,^^36^^,^^37^^,^^41^ Less frequently, admissions were arranged in response to patients’ needs or wishes,^36^^–^^38^ or patient and informal caregiver distress and anxiety.^39^^,^^42^

Circumstances pertaining to structures and processes included lack of systems to ensure continuity of care and/or limited access to patient information;^1^^,^^10^^,^^15^^,^^28^^,^^32^^,^^36^^,^^38^ lack of flexibility in OOH service provision;^28^^,^^35^^,^^36^ unavailability or limited capacity of alternative community services;^15^^,^^38^ lack of time to assess and address needs;^38^^,^^42^ and complexities of workforce management.^1^ Provider-related circumstances included poor communication and lack of training;^1^ unfamiliarity with patients;^28^^,^^35^^,^^37^^,^^42^ and feelings of professional underperformance or fears of disciplinary repercussions if hospital admissions were not instigated.^15^ Policy-related circumstances were associated with lack of advance care planning^15^^,^^28^^,^^42^ or concerns about timely access to anticipatory medications.^28^

Clinical factors related to assessment or diagnosis, symptom management, and precautionary admissions. Admissions were predominantly associated with treatment of pain or other physical symptoms including rapid deterioration in a patient’s condition27–31,33,34,36,37,40 and complications or failure of initial treatment.^29^^,^^30^^,^^34^ Other reported circumstances related to: clinical or diagnostic uncertainty;^15^^,^^30^ complexity of the situation;^39^ need for further investigation;^37^ abnormal laboratory results;^29^ or as a precautionary measure.^31^

### Processes of OOH hospital admissions at the end of life

Limited evidence is available for the processes review question from seven publications,^1^^,^^12^^,^^26^^,^^35^^,^^39^^,^^43^^,^^44^ with considerable variation in the level of detail provided. Processes were described as a chain of communication including a series of steps (triaging) and involving a number of different providers, at times using NHS pathways algorithms^12^ or agreed protocols.^44^ These processes varied considerably between services. Calls were answered by nonclinical call handlers,^1^^,^^12^^,^^44^ experienced specialist nursing staff,^39^ or other healthcare professionals.^1^ Triaging was by initial telephone consultation with a clinician,^12^ by forwarding to specialist advice^39^ or call back from a healthcare professional.^44^ Hospital admissions were arranged at all triage stages: on initial call to a call handler, after clinician phone consultation, or following a home visit.^44^

### Mechanisms of OOH hospital admissions at the end of life

In the 23 publications that indicated the services arranging admissions,^1^^,^^10^^,^^12^^,^^15^^,^^26^^,^^27^^,^^29^^–^^36^^,^^38^^–^^46^ OOH GP services were most often reported1,^10^^,^^26^^,^^27^^,^^29^^–^^33^^,^^35^^,^^36^^,^^38^^,^^40^^–^^42^^,^^44^^,^^45^ alongside ambulance 999 calls,^1^^,^^10^^,^^15^^,^^30^^,^^35^^,^^36^^,^^38^^,^^40^^,^^41^^,^^45^ NHS 111/NHS 24/other telephone advice lines,^1^^,^^35^^,^^38^^–^^42^^,^^46^ community nursing teams,^1^^,^^10^^,^^35^^,^^38^^,^^40^^,^^42^ palliative care teams^1^^,^^26^^,^^40^ and unspecified ‘unscheduled primary care’.^12^^,^^34^^,^^43^

### Service providers arranging hospital admissions at the end of life

In the 17 publications that indicated the practitioners arranging admissions,^1^^,^^10^^,^^12^^,^^15^^,^^27^^,^^29^^–^^32^^,^^36^^,^^37^^,^^39^^,^^40^^,^^42^^–^^44^^,^^46^ OOH GPs were most often reported,^10^^,^^27^^,^^29^^–^^32^^,^^37^^,^^40^^,^^42^ followed by community/palliative care nurses,^15^^,^^27^^,^^29^^,^^39^^,^^40^^,^^42^^,^^46^ call handers/999 operators,^1^^,^^12^^,^^36^^,^^42^^,^^44^ paramedics and ambulance staff,^10^^,^^15^^,^^27^^,^^30^ and unspecified ‘OOH clinicians’.^12^^,^^31^^,^^32^^,^^36^^,^^43^^,^^44^

### Patients receiving end-of-life care admitted to hospital OOH

Publications presented either generic information concerning patients admitted to hospital,^10^^,^^27^^,^^31^^,^^34^^,^^45^ data describing palliative and end-of-life care patient populations,^36^^,^^38^^,^^43^ or focused on patients with cancer.^1^^,^^10^^,^^15^^,^^27^^,^^29^^,^^31^^,^^40^^,^^42^^,^^44^^–^^46^ Few publications referred to non-cancer patients^46^ including those with chronic obstructive pulmonary disease and dementia,^10^^,^^15^ advanced dementia,^41^ and frailty.^37^

### Frequency of OOH hospital admissions at the end of life

The review question relating to frequency of OOH hospital admissions had the least evidence available, with diverse contexts, study designs, and populations studied. Fifteen publications presented widely varying frequencies of OOH end-of-life hospital admissions,^10^^,^^12^^,^^27^^–^^35^^,^^39^^,^^43^^–^^45^ ranging from 2%^44^ to 69% of patients.^28^ Limited conclusions can be drawn from the scarce and heterogeneous literature.

## DISCUSSION

### Summary

The literature addressing OOH end-of-life care hospital admissions in the UK is largely unfocused and limited by the heterogeneity of the evidence presented. Available data indicates that admissions are initiated in relation to informal caregiver and patient distress, discontinuity (or unavailability) of services and/or access to patient information, and symptom management issues. Hospital admissions are arranged by a variety of OOH services and providers, most prominently OOH GPs. The limited evidence focuses largely on cancer populations and reported admission rates varied greatly between studies.

### Strengths and limitations

To the authors’ knowledge, this is the first systematic review of the factors leading UK OOH services to admit patients receiving end-of-life care to hospital. Initial database searches were adapted to ensure conciseness and concreteness. Clearly defined criteria for study selection and explicit methods for data extraction and synthesis reduced biases and offered a transparent and replicable process. The patient and public involvement advisory group assisted in refining the research questions and interpretation of findings.

The review was hindered by inconsistent definitions of patients receiving palliative care and end-of-life care, and often heterogeneous patient populations in the included studies. The depth of narrative synthesis was limited by the focus of the studies identified and the nature of available evidence, which addressed diverse aspects of OOH provision either generically or indirectly. The focus of many studies on patients with cancer may not be generalisable to non-cancer populations that have been little studied to date.

### Comparison with existing literature

The circumstances identified in this review as leading OOH providers to instigate hospital admissions are well-known and documented. A UK 2001 report on OOH community palliative care identified challenges in service provision, communication, patient and carer support, and medical provision, including access to drugs, equipment, and specialist advice.^47^ These issues are also echoed in international research on the challenges for home-based OOH end-of-life care provision^1^^,^^26^^,^^48^^–^^50^ and explanations for unplanned hospital admissions for patients receiving end-of-life care.^51^^–^^53^ In this context of difficult end-of-life care OOH provision, UK and international studies have identified hospital admissions to be an invaluable resource, readily available at all times of the day and night, and offering a safe solution to issues that may be difficult to resolve in the community at short notice.^7^^,^^15^ Not yet addressed in the literature is why hospital admissions are at times not sought in the circumstances described. It is unclear, for example, whether any single issue or combination of issues is particularly significant for initiating (or protective of) OOH clinicians seeking hospital care for patients receiving end-of-life care.

### Implications for research and practice

This review provides evidence as to why issues experienced during OOH may lead to end-of-life hospital admissions (circumstances), how such admissions occur (processes), and by whom they are instigated (mechanisms). Importantly, although the findings may be unsurprising to many clinicians and end-of-life care researchers, this review highlights significant gaps in the evidence. Knowledge on how the identified factors interact with each other (for example, how circumstances may affect processes or how different mechanisms may respond to different circumstances) is currently lacking. Also lacking is evidence of effective interventions to improve care to prevent potentially avoidable end-of-life hospital admissions.^54^

The issues highlighted are pertinent to end-of-life care provision at all times of the day and night, although they seem to be particularly acute when they occur OOH. What the current review suggests is that, although OOH end-of-life care can often be readily resolved by hospital admissions, it comes with multiple challenges that appear to be widespread and systemic. Some of these challenges might be prevented by action in-hours^1^ or better management of unscheduled care episodes within the community leading to reduced hospital admissions, which is what most recent empirical evidence seems to suggest.^55^ Bearing in mind, however, that the OOH period comprise the majority of the week, service managers, commissioners, and policymakers need to continue to strive for integrated and comprehensive approaches to end-of-life care, 24 hours a day, 7 days a week.^56^^,^^57^
